# Supplementary material for: Follicle-Stimulating Hormone Alleviates Ovarian Aging by Modulating Mitophagy- and Glycophagy-Based Energy Metabolism in Hens
Source: Cells. 2022 Oct 18;11(20):3270. doi: 10.3390/cells11203270 (PMC9600712; doi:10.3390/cells11203270)
Supplement: Supplementary file 1 [file cells-11-03270-s001.zip › cells-1925114-supplementary.pdf]

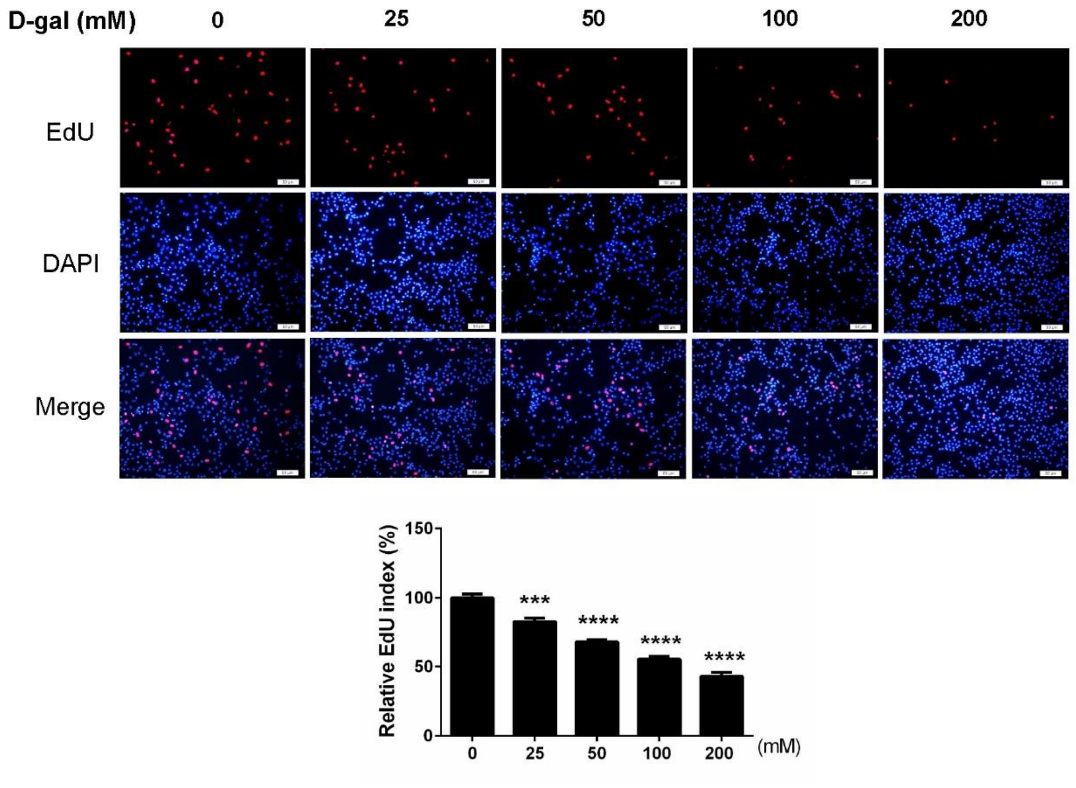

Figure S1: The effect of D-gal on cell proliferation in SYF-GCs of D280 hens. EdU labeled-proliferative GCs from control and D-gal-treated groups with different concentrations (0, 25, 50, 100, 200 mM). Scale bar: 50 µm. ***$P < 0.001$, which represents the difference between the groups in which the GCs were treated with D-gal of different concentrations for 24 h compared to control.

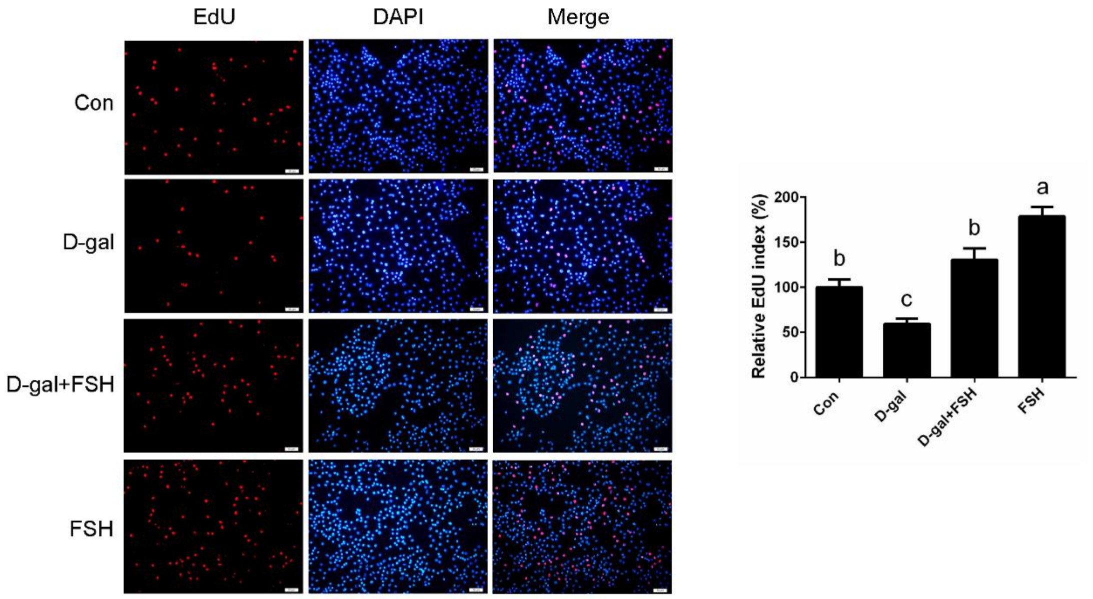

Figure S2: The effect of FSH on cell proliferation in D-gal-induced senescent GCs. SYF-GCs

of D280 hens were cultured with D-gal (200 mM) for 24 h, and then incubated with FSH (0.01 IU/mL) for another 24 h. EdU assay in GCs from the control GCs, senescent GCs (treated with 200 mM D-gal for 24 h), senescent GCs (treated with 200 mM D-gal for 24 h) with FSH treatment (0.01 IU/mL for another 24 h), and FSH treatment alone GCs (0.01 IU/mL). Scale bar: 50 μm. Different lowercase indicated significant differences ($P < 0.05$).
